# Young Chinese female body skin pigmentation map: A pilot study

**DOI:** 10.1111/srt.13567

**Published:** 2024-01-07

**Authors:** Yuqing Han, Yexiang Zhang, Benyue Li, Jie Yang, Yunji Qi, Qi Liu, Sisi Chang, Hua Zhao, Yao Pan

**Affiliations:** ^1^ Department of Cosmetics, School of Light Industry Science and Engineering Beijing Technology and Business University Beijing China; ^2^ Beijing Key Laboratory of Plant Research and Development Beijing China; ^3^ Shandong Huawutang Biotechnology Co., Ltd Jinan China; ^4^ Beijing EWISH Testing Technology Co., Ltd Beijing China

**Keywords:** body mapping, body skin, ITA°, MI, pigmentation distribution, skin pigmentation

## Abstract

**Background:**

Most studies have discussed variations in facial skin colour based on age, gender, and anatomical site within a specific ethnic group. However, skin pigmentation on the body is also a concern for many people.

**Aim:**

The aim of this study is to gather baseline data for Chinese young females, conduct a comprehensive assessment of body skin pigmentation, and create a body skin pigmentation map.

**Method:**

Individual type angle (ITA°) was registered by CL 400 and melanin index (MI) was registered by MX 18 in 100 body points of 20 Chinese females. A total of 12,000 measurements were recorded.

**Result:**

Our results showed significant differences among the symmetrical points on both sides of the body, including the clavicle, inner wrists, groin, inner ankle, elbow, armpit, waist side, the space between the thumb and index finger, instep, back shoulder, and popliteal space. Of all the points tested on the body, the points with the most severe skin pigmentation were the back of the neck, the heel, the elbow, and the popliteal space.

**Conclusion:**

This is the first comprehensive study of skin pigmentation conducted on the human body. In young Chinese women, the points with the most severe skin pigmentation were the back of the neck, heels, elbows, and the popliteal space.

## 1 INTRODUCTION

Skin is the largest and most easily observed organ of the human body. It plays a significant role in assessing human age, beauty, attractiveness, and health.^1^ The colour of skin is determined by four pigments: melanin, carotene, oxygenated haemoglobin, and reduced haemoglobin. Melanin (eumelanin and pheomelanin) gives skin the brown or black pigment that defines our skin colour. The other pigments play a lesser role in skin colour.^2^ Skin pigmentation arises through a specific and complex mechanism that causes the accumulation of melanosomes in keratinocytes, which serves to protect the skin from solar irradiation. Various nonspecific intrinsic factors, such as hormonal environment and inflammation, as well as extrinsic factors, such as solar irradiation, environmental pollution, and drugs, can modulate genetically determined melanin levels.^3^

Noninvasive objective measurements of skin colour can help prevent subjective bias caused by expert assessments. The two most frequently employed techniques are narrowband reflectance spectrophotometry and tristimulus colorimetry. In narrow‐band reflectance spectrophotometry, differences in red and near‐infrared light absorption and reflection of haemoglobin and melanin are used to measure the vascularization (erythema) and pigmentation (melanin) of the skin. In tristimulus colorimetry, colour is represented by the L*a*b* system, which was developed by the Commission Internationale d'Eclairage to provide a uniform description of all colours that are visible to the human eye. The L* value represents brightness on a spectrum from black to white, the a* value represents the value on a spectrum from red to green, and the b* value represents the value on a spectrum from blue to yellow, thus generating a quantitative description of colour. The individual type angle (ITA°) and hue angle, calculated from L* and b*, are good for detecting subtle variations in skin colour within the same ethnic group.^4^, ^5^ Their application extends to dermatology, aesthetic medicine, and cosmetics.^5^

Studies have discussed variations in facial skin colour based on age, sex, and anatomical site within a specific ethnic group, but skin pigmentation on the body is also a concern for many people.^6^ Since proper evaluation of body skin pigmentation requires a standardized and objective system, we aimed to gather baseline body skin colour data of young Chinese females, conduct a comprehensive assessment of the distribution of body skin pigmentation, and create a body skin colour map of this demographic.

## 2 METHODS

### 2.1 Subjects

This study was conducted with 20 healthy Chinese female subjects who had acne or acne marks on the back. The subjects were aged between 20 and 29, with a height of 158 cm∼168 cm and BMI of 18.5∼23.9. We selected subjects with an ITA° on the back of 20°−41°, which was defined according to the *Safety and Technical Standards for Cosmetics*.^7^ Subjects with outdoor work experience, BMI greater than 27 or skin disorders such as psoriasis, vitiligo, or ichthyosis were excluded.

This study followed the principles of the Declaration of Helsinki and Good Clinical Practice (GCP) regulations. The study protocol was approved by the Shanghai Ethics Committee for Clinical Research. All procedures were explained in detail, and written informed consent was obtained from all 20 subjects.

### 2.2 Study design

Before the measurement, the subjects rested for at least 30 min in a room at 20°C and 50% relative humidity, respectively. ITA° was measured with a Colorimeter CL 400 (Courage & Khazaka, Germany). The melanin index (MI) was measured using a Mexameter MX 18 (Courage & Khazaka, Germany).

**FIGURE 1:**
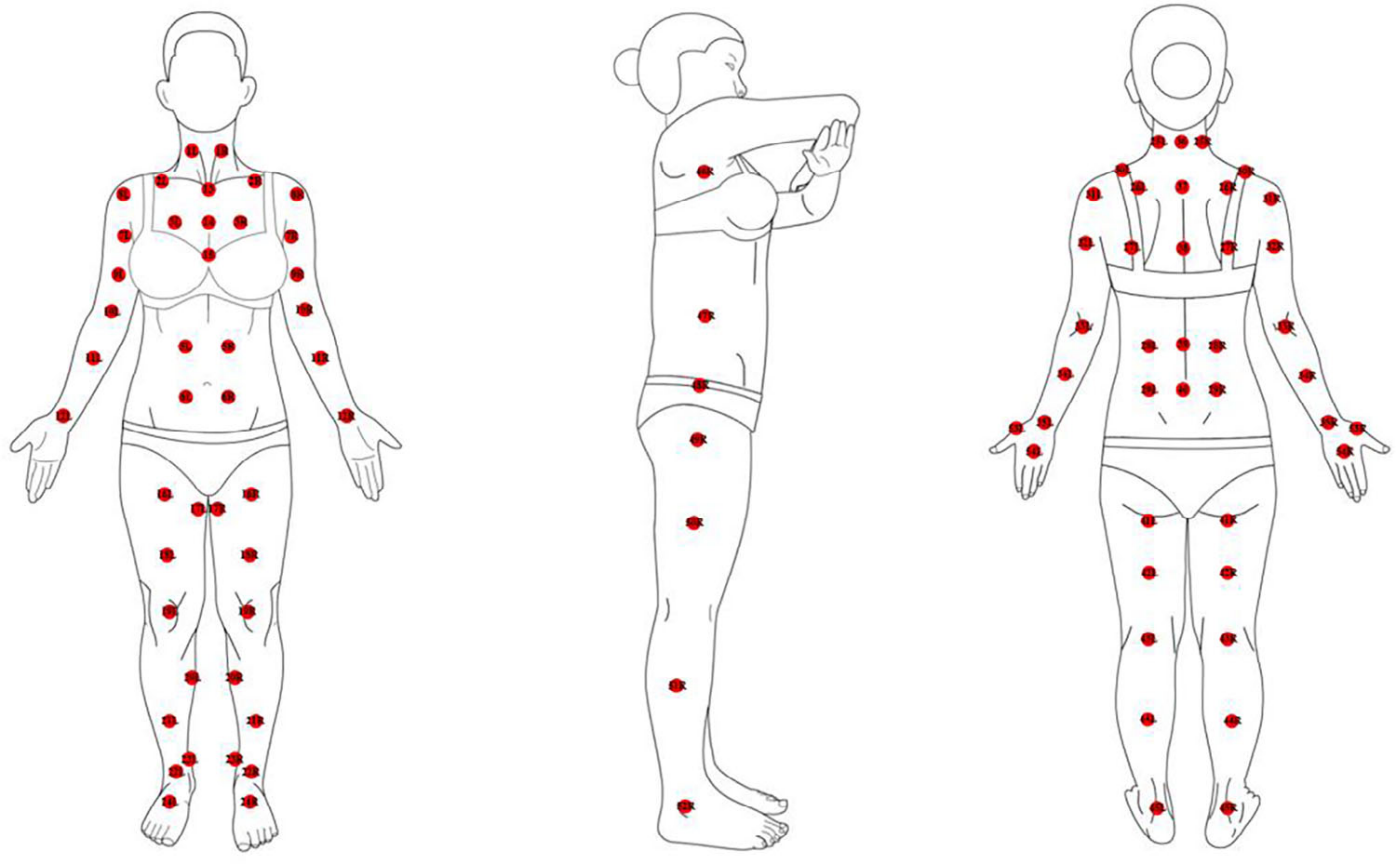
Schematic of body measurement points. One hundred measurement points were labeled on the front, side, and back of the human body schematic, respectively. Among them, points 1–12, 16–35, and 41–54 were symmetrical points, while points 13–15 and 35–40 were midline points. Each point was repeated three times to minimize errors.

One hundred points on the skin were measured, including on the front, back, and sides of the body (Figure 1 and Table 1). The specific points are shown in Figure 1. Each point was measured three times to avoid any erroneous values, and the mean values were taken. A total of 12,000 measurements were recorded for the 20 subjects.

**TABLE 1:** Details of body measurement points.

Point No.			Anatomical site
Front side	Point 1 left	Point 1 right	Neck
	Point 2 left	Point 2 right	Clavicle
	Point 3 left	Point 3 right	Upper chest
	Point 4 left	Point 4 right	Lower chest
	Point 5 left	Point 5 right	Waist
	Point 6 left	Point 6 right	Abdomen
	Point 7 left	Point 7 right	Inner upper arm
	Point 8 left	Point 8 right	Top of upper arm
	Point 9 left	Point 9 right	Middle of upper arm
	Point 10 left	Point 10 right	Fossa cubitalis
	Point 11 left	Point 11 right	Middle of forearm
	Point 12 left	Point 12 right	Inner wrists
	Point 13		Middle of clavicle
	Point 14		Middle of upper chest
	Point 15		Middle of chest
	Point 16 left	Point 16 right	Upper thigh
	Point 17 left	Point 17 right	Groin
	Point 18 left	Point 18 right	Middle of thigh
	Point 19 left	Point 19 right	Knees
	Point 20 left	Point 20 right	Inner calf
	Point 21 left	Point 21 right	Anterior calf
	Point 22 left	Point 22 right	Anterior ankle
	Point 23 left	Point 23 right	Inner ankle
	Point 24 left	Point 24 right	Instep
Back side	Point 25 left	Point 25 right	Neck
	Point 26 left	Point 26 right	Upper shoulder blade
	Point 27 left	Point 27 right	Lower shoulder blade
	Point 28 left	Point 28 right	Lumbar vertebra
	Point 29 left	Point 29 right	Flank
	Point 30 left	Point 30 right	Shoulder
	Point 31 left	Point 31 right	Top of upper arm
	Point 32 left	Point 32 right	Middle of upper arm
	Point 33 left	Point 33 right	Elbow
	Point 34 left	Point 34 right	Middle of forearm
	Point 35 left	Point 35 right	Wrist
	Point 36		Middle of neck
	Point 37		Middle of shoulder blade
	Point 38		Median of back
	Point 39		Middle of lumbar vertebra
	Point 40		Middle of flank
	Point 41 left	Point 41 right	Lower hip
	Point 42 left	Point 42 right	Middle of thigh
	Point 43 left	Point 43 right	Popliteal space
	Point 44 left	Point 44 right	Middle of calf
	Point 45 left	Point 45 right	Heel
Lateral side	Point 46 left	Point 46 right	Armpit
	Point 47 left	Point 47 right	Waist
	Point 48 left	Point 48 right	Crotch
	Point 49 left	Point 49 right	Upper thigh
	Point 50 left	Point 50 right	Middle of thigh
	Point 51 left	Point 51 right	Middle of calf
	Point 52 left	Point 52 right	Lateral ankle
	Point 53 left	Point 53 right	The space between the thumb and index finger
	Point 54 left	Point 54 right	Opisthenar

After the measurements, the subjects completed a questionnaire about their daily behavioural habits and the Baumann skin type questionnaire (pigmentation).

### 2.3 Body skin pigmentation mapping

To visualize the data from the various body locations, 2D contoured pigmentation colour maps were computed using Python software with the matplotlib library. Each predefined position was located on the body template, and the X and Y coordinates were recorded. The colour map was generated based on the average measurements of 20 subjects. For this purpose, a circular map was created on the body template, with H representing the mean value of colour and X and Y representing the coordinates.

### 2.4 Statistical analysis

The data were analysed using IBM SPSS version. 22.0 and GraphPad Prism version. 7.0. Means and standard deviations (SD) are reported for continuous variables, while categorical variables are expressed as proportions. Statistical differences between symmetric points were analysed using the paired t test. The significance level was set at *p* < 0.05.

## 3 RESULTS

### 3.1 Characteristics of the subjects

A total of 20 healthy Chinese female subjects were included in this study. Their average age was 23.75 ± 3.18 years, average height was 1.63 ± 0.04 m, average weight was 51.13 ± 5.56 kg, and average BMI was 19.95 ± 1.45. All BMIs were within the normal range, as they were not malnourished, overweight, or obese. The mean back ITA° was 34.20° ± 5.79°, which fell within the standard range of 20° to 41°. The mean score on the Baumann Skin Type Questionnaire (BSTQ) score was 29.48 ± 5.26, indicating pigmented skin characteristics that correspond to Fitzpatrick's skin types III and IV. The above information indicates that the subjects were similar in all aspects (Table 2).

**TABLE 2:** Characteristics of the subjects.

	*N*	Mean	Standard deviation	Standard error	Minimum value	Maximum value	Median value	Variance	Range
Age	20	23.75	3.18	0.71	18.00	29.00	24.00	10.09	11.00
Height	20	1.63	0.04	0.01	2.00	2.00	1.62	0.00	0.00
Weight	20	53.13	5.65	1.26	47.00	65.00	52.00	31.94	19.00
BMI	20	19.95	1.45	0.32	19.00	23.00	19.49	2.10	4.00
Back ITA°	20	34.20	5.79	1.30	25.00	41.00	36.00	33.57	16.00
BSTQ score	20	29.48	5.26	1.18	20.50	39.00	29.25	27.67	18.50

### 3.2 Symmetry analysis

**FIGURE 2:**
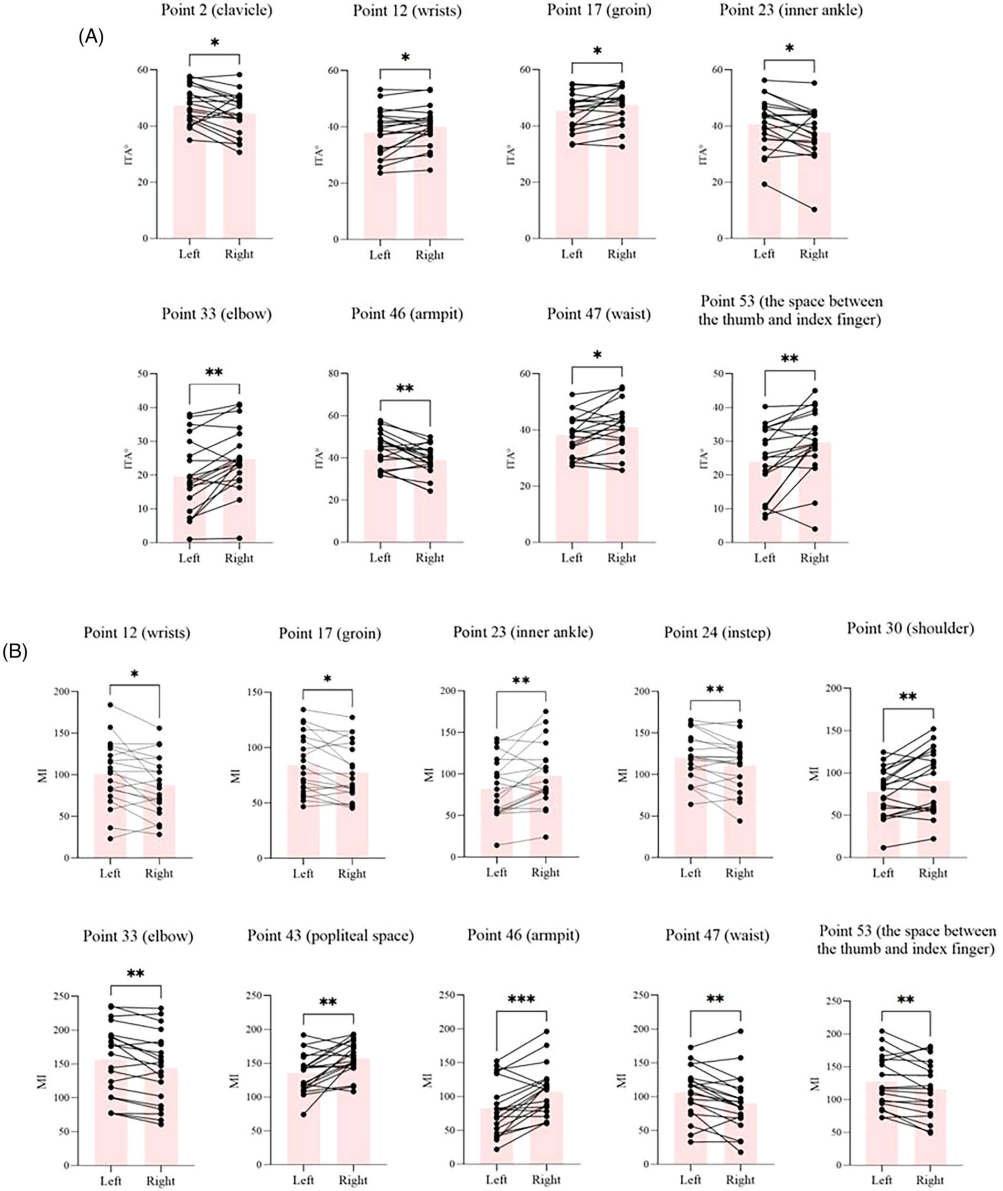
Paired t test results for symmetrical points in terms of (A) ITA° and (B) MI. Black dots represent measurements taken from symmetrical points on both the left and right sides. Black line segments represent two black dots from the same subject. The pink columns represent the average measurement of 20 subjects. *p < 0.05, **p < 0.01, and ***p < 0.001 compared as indicated.

Out of the 100 points tested, 92 points were part of a symmetrical pair on the left and right sides of the body. The exceptions were midline points 13–15 and 36–40, which were “solo” points down the middle of the body (Figure 1). The analysis revealed significant ITA° differences within eight symmetrical pairs: point 2 (clavicle), point 12 (inner wrists), point 17 (groin), point 23 (inner ankle), point 33 (elbow), point 46 (armpit), point 47 (waist side), and point 53 (the space between the thumb and index finger) (Figure 2A). Ten pairs differed in MI: point 12 (inner wrists), point 17 (groin), point 23 (inner ankle), point 24 (instep), point 30 (back shoulder), point 33 (elbow), point 43 (popliteal space), point 46 (armpit), point 47 (waist side), and point 53 (the space between the thumb and index finger) (Figure 2B).

### 3.3 Pigmentation severity ranking

**FIGURE 3:**
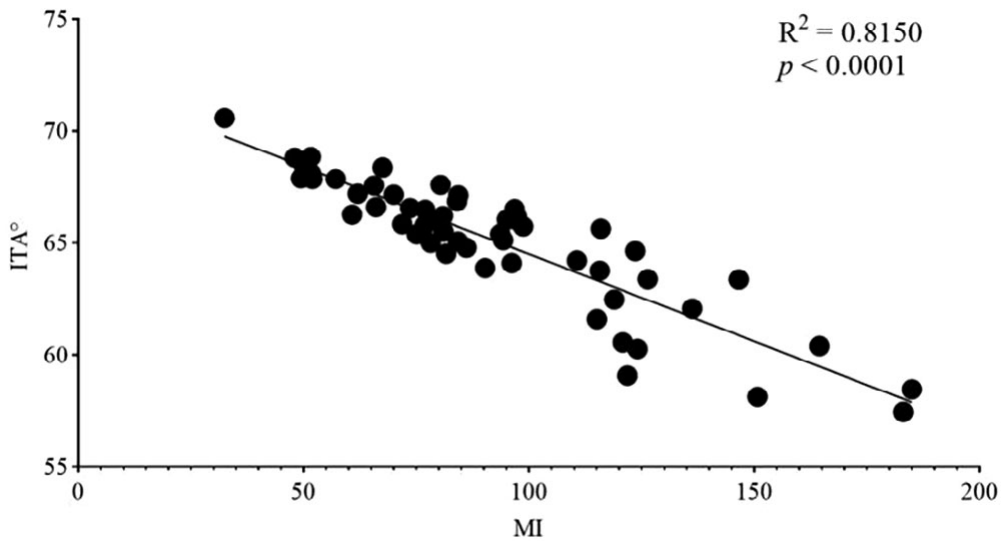
Correlation between MI and ITA°. Correlation analyses were performed using the mean values of MI and ITA. The mean values were calculated using three replicated measurements for the median points and six replicated measurements on both sides for the symmetrical points. Good correlation between MI and ITA°.

**FIGURE 4:**
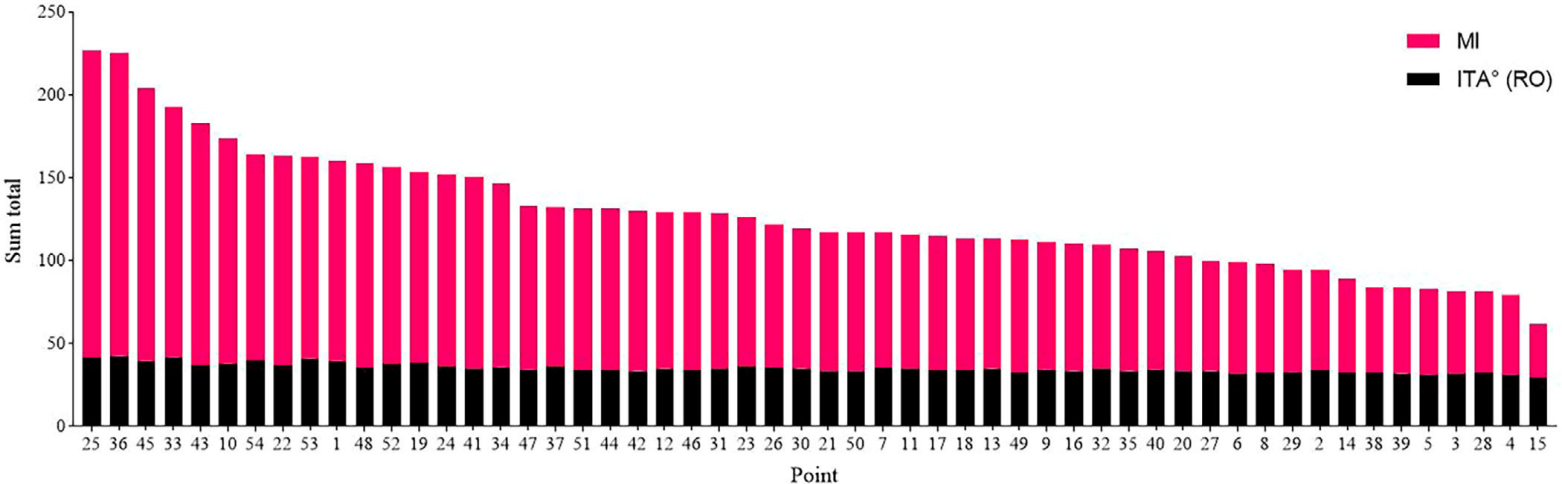
Ranking of points by body skin pigmentation. In this grouped stacked bar chart, black represents ITA° (RO) and red represents MI. The ITA° (RO) was added to the MI and then sorted by its sum.

There was a strong correlation between MI and ITA°, with an R^2^ value of 0.8150 (Figure 3). Since a higher ITA° represents a lighter skin tone and a higher MI represents a darker skin tone, we aligned the trends of the two by processing ITA° in reverse order. We named the reversed value ITA° (RO). The directional trend of ITA° (RO) was consistent with that of MI, both indices indicating darker skin colour with higher values. The data from all points were measured 6 times in parallel for symmetrical points and 3 times in parallel for the midline points, and we calculated the averages of these repeat measurements. The average ITA° (RO) was then added to the average MI, and the sum was ranked from highest to lowest (Figure 4). A larger sum represented more severe pigmentation at that point. As depicted in Figure 3, the five points exhibiting the most pronounced pigmentation were point 25 (back neck), point 36 (back middle of neck), point 45 (heel), point 33 (elbow), and point 43 (popliteal space), while the five points with the lightest pigmentation were point 15 (middle of chest), point 4 (lower chest), point 28 (lumbar vertebra), point 3 (upper chest), and point 5 (waist). We also found that the changes in MI were more intuitive than the changes in ITA° (RO), so we used MI to draw the map of body skin pigmentation.

### 3.4 Questionnaire results

After completing the data collection, the subjects were asked to fill in a behavioural questionnaire. The specific results of the questionnaire are presented in Figure 4. All subjects were employed in indoor occupations and reported a low frequency of outdoor exercise (10%). This meant that the subjects had not been exposed to sunlight for long times. A high proportion (95%) regularly used sunscreen products. They were most concerned about skin pigmentation on their knees (30%) and hands (45%) but did not pay attention to sun protection on the body overall (fewer than 10% answered yes for all areas). This indicates that the subjects were aware of the need for sun protection but did not go to much effort to protect their bodies from the sun. Body lotion (85%) and scrub (65%) were used more frequently, indicating a high level of skincare awareness. Eighty percent of the participants preferred a bralette over an underwire. Outfit choices mainly consisted of short‐sleeved shirts (80%), trousers (70%), shorts (55%), long dresses (50%), and wrist jewellery (85%). Summer hairstyles were mainly longer than shoulder length (55%), and 100% tied their hair up. This means that the back of every participant's neck got a relatively high amount of sun exposure. Sandals were the most popular footwear, accounting for 55% of the subjects. Some 80% of the participants reported experiencing foot abrasions, primarily on their heels.

### 3.5 Body skin pigmentation mapping

A map of body skin pigmentation was drawn using the MI values (Figure 6). The purpose of the figure was not to reflect any true skin colour but simply to show the differences in pigmentation between the different points, so we chose exaggerated colours for the map. Darker colours represent more intense skin pigmentation. Figure 6A depicts the front side of the body, point 10 (fossa cubitalis), point 19 (knees), and point 24 (instep) exhibiting the most pronounced skin pigmentation. Figure 6B represents the posterior view of the body. The areas with the most severe pigmentation were point 25 (neck), point 36 (median of the neck), point 33 (elbow), point 43 (popliteal space), and point 45 (achilles tendon). Figures 6C and 6D depict the body's sides, where the areas with the highest pigmentation were point 46 (armpit), point 48 (crotch), and point 52 (lateral ankle). The lightest points of pigmentation were concentrated on the upper body, which is completely covered by clothing.

## 4 DISCUSSION

In this study, we conducted the first comprehensive analysis of body skin pigmentation in Chinese women aged 20–29 years. In previous studies related to skin pigmentation, either only the face or only certain parts of the body have been assessed.^8^, ^9^, ^10^, ^11^, ^12^, ^13^, ^14^, ^15^ Our results showed significant differences in ITA° between several pairs of left/right symmetrical points. These points included point 2 (clavicle), point 12 (inner wrists), point 17 (groin), point 23 (inner ankle), point 33 (elbow), point 46 (armpit), point 47 (waist side), and point 53 (the space between the thumb and index finger) (Figure 2A). Significant left/right differences in MI were found at point 12 (inner wrists), point 17 (groin), point 23 (inner ankle), point 24 (instep), point 30 (back shoulder), point 33 (elbow), point 43 (popliteal space), point 46 (armpit), point 47 (waist side), and point 53 (the space between the thumb and index finger) (Figure 2B). MI correlated well with ITA° (Figure 3). Among all the points tested on the body, the five points that had the darkest skin pigmentation were point 25 (back neck), point 36 (back middle of neck), point 45 (heel), point 33 (elbow), and point 43 (popliteal space). Point 15 (middle of chest), point 4 (lower chest), point 28 (lumbar vertebra), point 3 (upper chest), and point 5 (waist) were the five lightest points on the body (Figure 4). We considered the MI differences to be more pronounced than the ITA° differences and established the map based on MI (Figure 6).

It's important to note that both ITA° and MI lack the ability to assess skin texture, they are designed for measuring skin colour. Treesirichod et al.^16^ and Bitterman et al.^17^ dispute the question of whether skin texture affects skin pigmentation. We agree with Bitterman et al ^17^ that, during actual measurements, we found that parameters taken from rough areas of the skin texture were not as stable as those taken from flat areas of the skin. Therefore, we chose to minimize the inclusion of areas with rough skin texture. Instead, we utilized the parameters obtained from the surrounding areas with relatively flat skin and calculated the average value to substitute the rough skin texture points. Examples include point 19 (knees) and point 33 (elbow).

**FIGURE 5:**
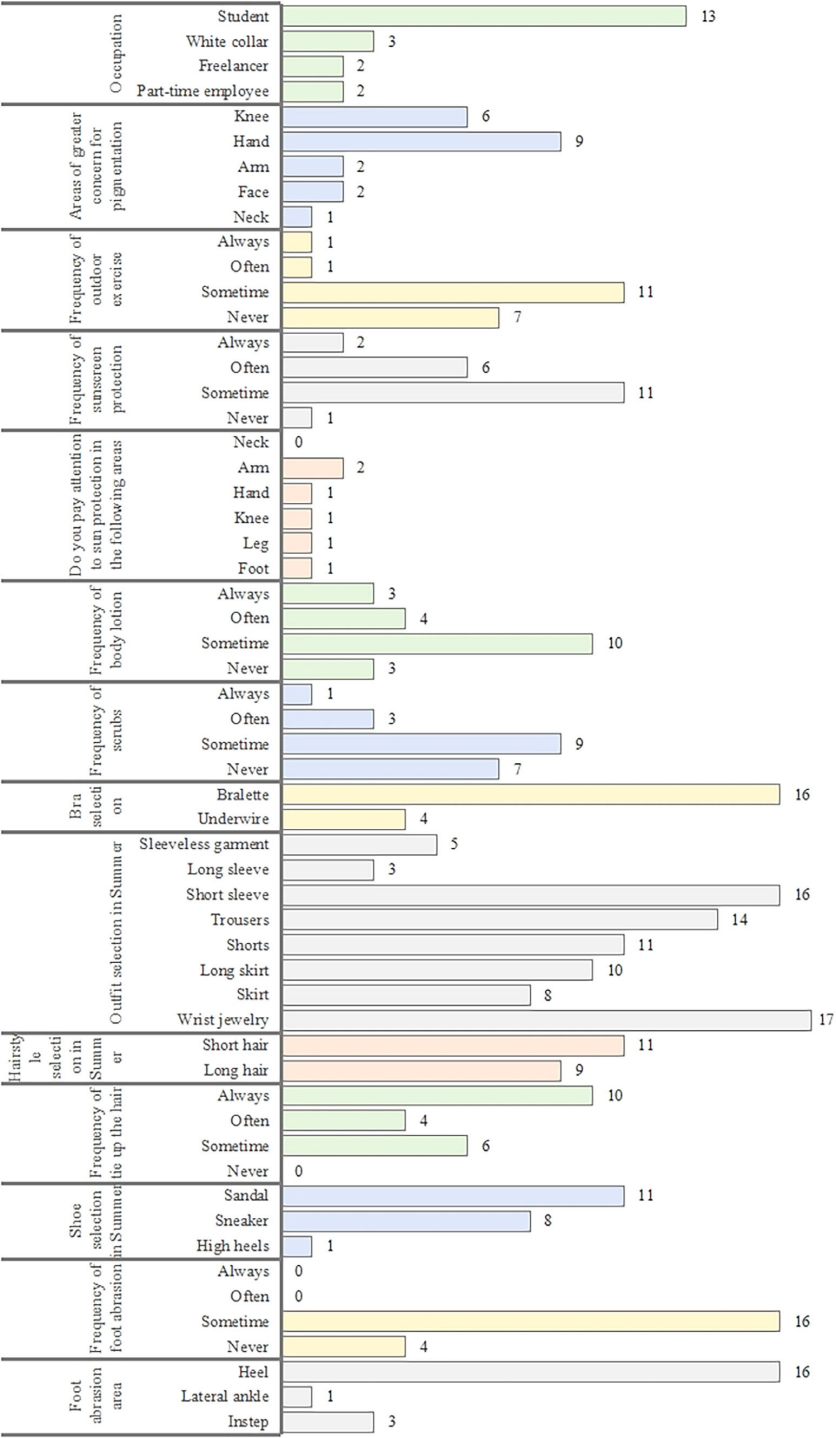
Questionnaire results. The vertical text on the left side represents the topic focus, while the horizontal text in the middle corresponds to the options related to the topic. The bar chart and numbers on the right side represent the number and percentage of people who selected each option.

The points of left/right MI differences were not all the same as the points of left/right ITA° differences (Figure 2). Significant differences were found in both ITA° and MI for the following points: point 12 (inner wrists), point 17 (groin), point 23 (inner ankle), point 33 (elbow), point 46 (armpit), point 47 (waist side), and point 53 (the space between the thumb and index finger). Point 2 (clavicle) was only significantly different in ITA°, while point 24 (instep), point 30 (back shoulder) and point 43 (popliteal space) were only significantly different in MI. This phenomenon has also been found in previous studies by our research group,^9^ and it was found in the study by Linde et al.^18^ We speculate that this might be a systematic error caused by the different operating principles of the two instruments. We measured ITA° using the CL 400, which works on the L*a*b* system. MI was measured by the MX 18, which works on narrow‐band reflectance spectrophotometry. This method utilizes differences in red and near‐infrared light absorption and the reflection of haemoglobin and melanin to assess the vascularization (erythema) and pigmentation (melanin) of the skin. ITA° is more susceptible to being skewed by ambient light than MI.^4^, ^5^ Point 2 (clavicle) was an apophysis. Point 24 (instep), point 30 (back shoulder), and point 43 (popliteal space) were on smooth parts of the skin. Such conditions can affect the refraction of light, resulting in inaccuracies in the final measurement. Although the results of the paired t tests were not entirely consistent, the overall correlation between ITA° and MI was good (Figure 3), which was consistent with the findings of Wilkes et al.^14^ Excessive UV exposure can cause skin pigmentation. Published studies clearly demonstrate the process of skin pigmentation after exposure to UVA or UVB rays.^19^, ^20^ According to the questionnaire results, most of our subjects neglected sun protection (Figure 5). Point 2 (clavicle), point 24 (instep), and point 43 (popliteal space) are susceptible to sunlight. As a result, skin pigmentation is more likely to occur in these areas due to sun exposure. Hormones have also been linked to skin pigmentation.^21^ We inferred that the skin pigmentation at point 46 (armpit) might be most likely hormone‐induced, but further research is needed to determine why there was a significant difference between the left and right sides. Besides sun exposure and hormones, there is one factor that is often overlooked: mechanical friction. Repetitive mechanical friction over a prolonged period can also result in skin pigmentation and may even lead to dermatoses.^22^ We believe that points 12 (inner wrists), 17 (groin), 23 (inner ankle), 33 (elbow), and 53 (the space between the thumb and index finger) are areas of apophysis or areas subjected to prolonged mechanical friction. We also speculate that there might have been a significant difference in skin pigmentation between certain pairs of points due to unequal stress.

To harmonize the trends of ITA° and MI, we processed ITA° in reverse order (ITA°(RO)). Ranking the sum of ITA° (RO) and MI yielded a clear graph of skin pigmentation at all the points (Figure 4). The five lightest points were point 15 (middle of chest), point 4 (lower chest), point 28 (lumbar vertebra), point 3 (upper chest), and point 5 (waist), all of which were areas on the front of the body covered by clothing. Surprisingly, point 15 (middle of chest) and point 4 (lower chest) were nearly free of pigmentation. Although mechanical friction can lead to skin pigmentation,^22^ 80% of the subjects in the questionnaire said they preferred bralettes (Figure 5), which cause less friction than underwires. Point 15 (median of the chest), point 4 (lower chest), point 28 (lumbar vertebra), point 3 (upper chest), and point 5 (waist) were all areas of the body that are not exposed to friction or sunlight. As a result, these points tend to have the mildest skin pigmentation. However, point 25 (back neck), point 36 (back middle of neck), point 45 (heel), point 33 (elbow), and point 43 (popliteal space) had the most pigmentation of all. Considering their deconstructed parts, point 25 (back neck), and point 36 (back middle of neck) may not have been protected from the sun enough. In the questionnaire, only 5% of the subjects cared about skin pigmentation on the neck, and none of them paid attention to sun protection on the neck area (Figure 5). The results for point 45 (heel) may be related to shoe friction. In the questionnaire, 55% chose sandals, 40% chose sneakers and 5% chose high heels as their footwear of choice. Eighty percent of the participants reported experiencing occasional abrasion on their feet caused by their shoes, with 80% of the affected area being on the heels (Figure 5). The results of point 33 (elbow) may be related to friction and dressing habits. Eighty percent preferred wearing short sleeves, suggesting that the elbows are often exposed to the sun, and only 10% of the subjects cared about sun protection on their arms (Figure 5). Considering that the elbow is a bony prominence, it is quite normal for pigmentation to be more severe there than the surrounding skin. We speculated that the results of point 43 (popliteal space) might be caused by sun exposure or genetic factors^23^.

**FIGURE 6:**
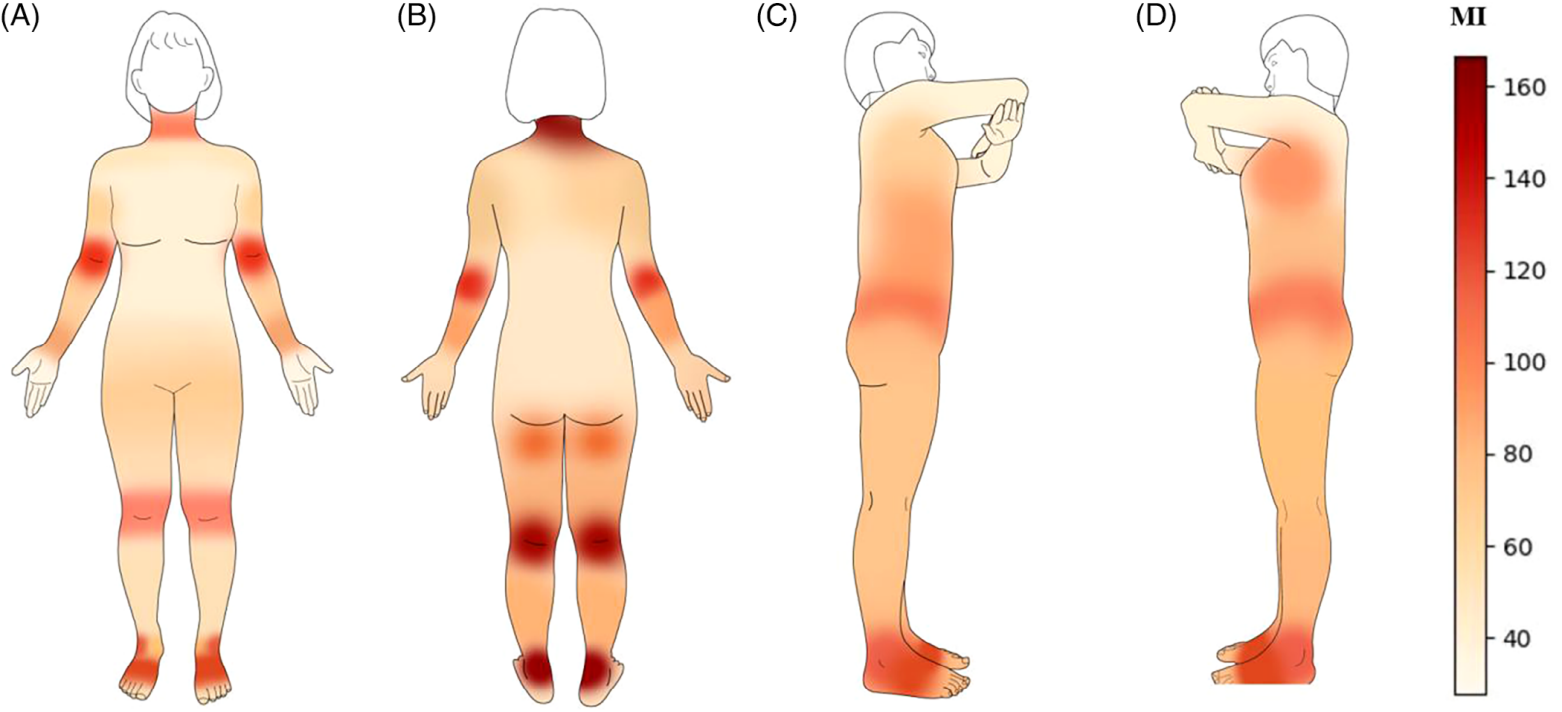
Body skin pigmentation map. The MI of 100 points will be displayed as a heat map in the model's diagram. The darker the colour, the more pronounced the skin pigmentation. (A) Front of the body, (B) Back of the body, (C) Right side of the body, and (D) Left side of the body.

Both ITA° and MI are highly correlated with the melanin content of the skin, but MI is specifically designed to measure the absorption spectra of melanin and haemoglobin, making it a potentially better indicator of melanin content than ITA.^24^ ITA° correlates with skin hydration, which varies from site to site, which may also lead to bias in ITA° measurements.^25^ As seen in Figure 4, the change in MI was more obvious than that in ITA°, so we chose to draw the body skin pigmentation map based on MI (Figure 6). As seen in Figure 6, the neck, arms, and thighs had more pronounced skin pigmentation. However, the skin on both the front and back of the upper body was fairer. This is quite normal, considering the summer dressing habits of Chinese women. There is a tendency to use inner forearm skin colour parameters to represent body skin colour in studies of the skin,^9^, ^11^, ^14^ and we found this feasible. The mean ITA° of the 100 points was 65.08, and the mean MI was 92.56. The ITA° and MI of the medial arm were 65.51 and 81.06, respectively, which were similar to the total mean. Choe et al.^15^ compared skin colour between the buttocks and forehead. Unlike their results, we found the average ITA° at point 41 (lower hip) was 65.63, while the range of ITA° on the buttock in their results was 27.01 to 45.35. This discrepancy might be due to the difference in the measurement point (below the hip vs. the buttock). Considering that 79% of the subjects in Choe's study were males and our study had only female subjects, we consider this difference acceptable. Hermanns et al.^11^ used MX16 to assess the MI of the inner arm, volar forearm, dorsal forearm, and forehead. Ultimately, the dorsal forearm was found to have the highest MI, followed by the forehead, the volar forearm, and the inner arm. Firooz et al.^12^ also measured parameters in several parts of the body, finding the neck the darkest, followed by the back, forearms, and legs, and lastly the palms. This is in general agreement with our findings (Figure 6).

As a pilot study, this study has some limitations. For example, factors such as sex,^15^ age group,^26^ season,^27^ and geographical area^28^ were not taken into account. In future studies related to body skin pigmentation in Chinese people, these factors should be thoroughly considered. Our study still has some reference value, and it may serve as a guide for future research on skin pigmentation across the body.

## 5 CONCLUSION

This is the first comprehensive study of skin pigmentation conducted on the human body. In young Chinese women, the sites with the most severe skin pigmentation were the back of the neck, heels, elbows, and the popliteal space. The sites with the least skin pigmentation were the median of the chest, lower chest, lumbar vertebra, upper chest, and waist. Importantly, the causes of skin pigmentation vary across different body parts. UV exposure, mechanical friction, hormones, genetics, and other factors all contribute to skin pigmentation. This study can help us understand the distribution pattern of body pigmentation in young Chinese women, which will aid in studying the causes of skin pigmentation, which will be important for the development of cosmetics.

## CONFLICT OF INTEREST STATEMENT

The authors have no conflicts of interest to declare.

## STATEMENT OF ETHICS

This study was conducted according to the principles of the Declaration of Helsinki and Good Clinical Practices (GCP) regulations. The study protocol was approved by Shanghai Ethics Committee for Clinical Research, approval number SECCR/2023‐116. All procedures involved in the study were explained in detail, and written informed consent was obtained from all 20 subjects.

## Data Availability

The datasets used and analysed during the current study available from the corresponding author on reasonable request.
